# Nanocomposite Film Containing Fibrous Cellulose Scaffold and Ag/TiO_2_ Nanoparticles and Its Antibacterial Activity

**DOI:** 10.3390/polym10101052

**Published:** 2018-09-20

**Authors:** Yanxiang Li, Jessica Tian, Chuanfang Yang, Benjamin S. Hsiao

**Affiliations:** 1 Key Laboratory of Green Process and Engineering, Institute of Process Engineering, Chinese Academy of Sciences, Beijing 100190, China; cfyang@ipe.ac.cn; 2 Department of Chemistry, Stony Brook University, Stony Brook, NY 11794, USA; jessicactian@gmail.com

**Keywords:** cellulose, Ag/TiO_2_ sol–gel, antibacterial activity, synergetic effect

## Abstract

Cellulose is a natural polymer that is widely used in daily life, but it is susceptible to microorganism growth. In this study, a simple sol–gel technique was utilized to incorporate the cellulose scaffold with Ag/TiO_2_ nanoparticles. The morphology and crystal structure of the as-prepared Ag/TiO_2_/cellulose composite film were characterized using scanning electron microscopy (SEM) and X-ray diffraction (XRD) methods. Antibacterial tests involving the use of Escherichia coli (*E. coli*) were carried out under dark and UV-light conditions to evaluate the efficiency of the Ag/TiO_2_/cellulose composite film in comparison with pristine cellulose paper and TiO_2_/cellulose composite film. The results indicated that the antibacterial activity of the Ag/TiO_2_/cellulose composite film outperformed all other samples, where the Ag content of 0.030 wt% could inhibit more than 99% of *E. coli*. This study suggests that finely dispersed nanocale Ag/TiO_2_ particles in the cellulose scaffold were effective at slowing down bacterial growth, and the mechanisms of this are also discussed.

## 1. Introduction

Cellulose is the most abundant biopolymer on Earth, with over 150 billion tons of biomass produced every year [1]. From a structural perspective, cellulose is a carbohydrate polymer generated from the repeating unit of β-d-glucopyranose molecules that are covalently linked through β-1,4-glucan [2]. Cellulose has a large number of hydroxyl groups (three per anhydroglucose (AGU) unit) on the repeating unit, which leads to extensive hydrogen bond networks that make it insoluble in common solvents. The unique structure and abundance of cellulose makes them a source of material with fascinating properties, including hydrophilicity, renewability, biodegradability, and biocompatibility. As a result, cellulose products are widely used in our daily life, such as basic cloths, foods, papers, pharmaceutics, and healthcare. Recently, they have also been considered in many advanced environmental and energy applications, such as for water treatment [3,4,5], solar cells [6,7,8], and supercapacitors [9,10,11], just to name a few.

Because of their unique chemical structure and properties, cellulose products are also ideal scaffolds for the growth of micro-organisms [12,13]. The reason why cellulose is more sensitive to bacterial colonization is because it is typically porous, hydrophilic, able to retain substantial water content, and also able to easily diffuse oxygen and nutrients throughout the scaffold, thus providing a perfect environment for bacterial growth. For practical applications, modification of cellulose with antibacterial properties is often necessary.

Silver ions (Ag^+^) and silver nanoparticles (Ag NPs) are well-known components for providing antibacterial activity [14,15,16], and many studies have reported the excellent antibacterial properties of polymer composites containing Ag NPs [14,15,16,17,18,19]. However, their relatively high manufacturing costs often limit their practical application. Titanium dioxide (TiO_2_) has also been demonstrated to have excellent antibacterial and photocatalytic properties under UV irradiation. This system has been extensively investigated due to their low-cost, non-toxic, and stable chemical and physical properties [20,21,22,23]. In brief, under UV irradiation, TiO_2_ can exhibit biocidal properties resulting from the generation of reactive oxygen species (ROS) [24]. The antibacterial activity of TiO_2_ thus depends on the rate of ROS formation with respect to the rate of recombination to the photo-induced electron-hole (H^+^/e^−^). Generally, the high recombination rate of photo-induced electron-holes and the wide band-gap energy can significantly limit the antibacterial and photocatalytic performance of TiO_2_.

The incorporation of Ag NPs was found to be able to narrow the band-gap energy of TiO_2_ and create some plasmonic processes at the surface of TiO_2_, thus enhancing its antibacterial activity [25,26,27,28,29]. Typically, there are three routes to fabricating Ag/TiO_2_ composites: the hydrothermal [30,31,32,33,34], photoreduction [23,35,36], and sol–gel [26,27,37] methods. The hydrothermal method can avoid the agglomeration of nanoparticles, but the required use of high temperature and high pressure to initiate the reaction often limits its value for industrial application. The photoreduction method involves the use of UV irradiation to deposit Ag NPs onto the TiO_2_ scaffold (e.g., nanosponges) in silver nitrate (AgNO_3_) solutions. However, the large-scale usage of UV irradiation on an industrial level can result in air pollution problems. In a way, the sol–gel synthesis represents a simple and efficient approach to fabricate nanoscale Ag/TiO_2_ composites as the method has been extensively demonstrated to produce uniform crystalline metal oxide thin films on the various substrates. For example, several studies dealing with the fabrication of TiO_2_ thin films on the cellulose substrates have been reported using the sol–gel method [20,38,39,40,41]. However, none has been reported to produce Ag/TiO_2_ composite nanoparticles directly from the cellulose scaffold, which was the purpose of this study.

In this work, we demonstrate a cost-efficient and environmentally-friendly method at room temperature to first prepare an Ag/TiO_2_ sol, followed by immobilization onto a cellulose scaffold (filter paper). The approach combines the sol–gel and dip-coating processes to fabricate Ag/TiO_2_/cellulose composite films (or papers). This approach has many advantages, including how: (1) it is a simple, green, and easily scalable process; (2) the process provides reduced manufacturing costs compared to the use of silver individually; (3) silver nanoparticles can be dispersed uniformly in the system without agglomeration; and (4) the system offers a synergetic effect by combining both silver and TiO_2_ antibacterial activities.

## 2. Materials and Methods

### 2.1. Materials

Titanium (IV)-n-butoxide (97%), nitric acid (70%), and ascorbic acid were purchased from Sigma Aldrich (St. Louis, MO, USA). Silver nitrate (AgNO_3_) was purchased from the Fisher Scientific Company (Hampton, NH, USA). Cellulose filter paper (Grade No 131, 100% alpha cotton) was purchased from Advantec (Taibei, Taiwan). All chemicals and cellulose substrate were used without further purification or pre-treatment.

### 2.2. Preparation of TiO_2_ Sol

In the typical procedure, 6 mL of Titanium (IV)-n-butoxide was first dissolved in 2 mL of isopropyl alcohol, and the solution was subsequently added drop-wise to a solution containing 2.0 mL of nitric acid and 200 mL of distilled H_2_O and vigorously stirred at room temperature. White precipitate was formed immediately upon each additional drop. The final mixture was vigorously stirred for 48 h, followed by aging for 48 h at room temperature to produce the TiO_2_ sol. The colloidal suspension of the TiO_2_ sol was a white-blue color, semi-transparent, but quite stable for over one month.

### 2.3. Preparation of Ag/TiO_2_ Sol

For every 10.0 mL of TiO_2_ sol, 31, 156, and 780 μL of 0.1 M AgNO_3_ solutions were used to produce theoretical 0.2%, 1.0%, and 5.0% Ag to TiO_2_ molar ratios of sols, respectively. The AgNO_3_ solution was also added drop-wise to the TiO_2_ sol in a dark container, while being vigorously stirred at room temperature. The suspension was stirred for 30 min before adding excess ascorbic acid (0.1 M) drop-wise, where the resulting sol was continuously stirred for another 30 min. The final Ag/TiO_2_ sol was orange-brown and semi-transparent.

### 2.4. Ag/TiO_2_/Cellulose Composite Film Fabrication

The cellulose substrate (commercial filter paper) was first submersed in the Ag/TiO_2_ sol for 30 s. The sol-saturated paper was then placed in a preheated oven at 65 °C for 5 min to remove some solvents, and then cured at 95 °C for 5 min to form TiO_2_ particles. After that, the impregnated paper was treated in boiling water for 2 h. During this process, the crystalline TiO_2_ particles became more perfect, and the unattached TiO_2_ particles were removed. Finally, the recovered Ag/TiO_2_/cellulose composite film was dried at 40 °C.

### 2.5. Scanning Electron Microscope (SEM)

The surface morphology of the Ag/TiO_2_/cellulose composite film was analyzed by a scanning electron microscope (SEM, FEG-SEM LEO 1550, Carl Zeiss, Germany) equipped with a Robinson backscattered electron detector and 10 eV Schottky field-emission gun. The instrument also contained an energy-dispersive spectroscopy (EDS) spectrometer (detector from EDAX and controller from Iridium Ultra software (iXRF)) to characterize the chemical composition.

### 2.6. Thermal Gravitational Analysis (TGA)

Thermal gravimetric analysis (TGA) was carried out on a TGA Q50 machine (TA, New Castle, DE, USA). The samples were run at a heating rate of 10 °C/min in the range of 20–700 °C under an air atmosphere.

### 2.7. X-ray Diffraction (XRD)

The X-ray diffraction (XRD) patterns were obtained using a D8 X-ray diffractometer (Bruker, Karlsruhe, Germany) with CuKα radiation. The chosen wavelength (λ) was 0.154 nm, which was generated by CuKα radiation at 40 kV and 40 mA using a Ni filter. Data collection was carried out using a flat holder in the Bragg-Brentano geometry (10°–60°, 5°min^−1^).

### 2.8. Zeta-Potential

The zeta-potential of the TiO_2_ sol was measured in triplicate with a Zetaprobe Analyzer^TM^ instrument (Colloidal Dynamics, St. Johns, FL, USA). This instrument consisted of a built-in titration set-up equipped with a pH electrode and ESA sensor probe. Before analyzing the sample, the pH electrode was calibrated using three different pH buffer standards (pH = 4.01, 7.01, and 10.01), followed by a standard titration solution. The ESA sensor was calibrated using the standard zeta probe polar solution (KSiW solution). Upon completion of the calibration test, the TiO_2_ sol was filled in the sample holder, where the ESA sensor was then introduced into the sample under magnetic stirring to analyze the zeta potential.

### 2.9. Transmission Electron Microscopy (TEM)

A piece of TiO_2_/cellulose film was vigorously stirred in water and subsequently sonicated to peel off the TiO_2_ NPs. The suspension was deposited on a carbon-coated copper grid and dried in air. The specimens were observed using a JEM 2100F transmission electron microscope (TEM, JEOL, Japan), operated at 200 kV.

### 2.10. Preparation of PBS

To prepare the *E. coli* sample for the antibacterial test, phosphate-buffered saline (PBS) buffer solution was prepared using the following procedure: NaCl (8.01 g), KCl (0.20 g), Na_2_HPO_4_ (1.14 g), and KH_2_PO_4_ (0.27 g) were mixed with 500 mL of distilled water in a beaker. The solution was then transferred into a 1.0 L volumetric flask, where more distilled water was added until the solution became 1.0 L. Finally, the PBS solution was stored in a large 1.0 L Pyrex jar and kept in a refrigerator.

### 2.11. Preparation of E. coli

*E. coli* was cultured in fresh lysogeny broth (LB), a nutritionally rich medium, overnight. The cells were centrifuged at 10,000 rpm for 4 min. The supernatant was decanted, and the cell pellet was re-suspended with PBS. The resulting cells were centrifuged and the supernatant was decanted again. This process was repeated one more time using PBS to separate the cells from the nutrient broth to prevent further cell growth. Once the supernatant was decanted for the third time, the cells, suspended in PBS, were transferred to a larger container, and PBS was added until the volume of the suspension was 360 mL.

### 2.12. Preparation of LB/Agar Plates

Peptone (5.0 g), yeast extract (2.5 g), NaCl (5.0 g), and agar (7.5 g) were first mixed with 250 mL of distilled water to form a homogeneous solution, where the final solution was diluted to a total volume of 500 mL. This solution was then transferred to a Pyrex jar, which was autoclaved at 121 °C in a liquid loading cycle. The final agar was cooled to approximately 55 °C before use.

To prepare the LB/agar plate, a layer of LB agar (~15 mL) was poured into a sterile petri dish. The plate was swirled in a circular motion to distribute the agar uniformly on the bottom of the dish. Each plate was cooled to room temperature, solidified (~20 min), and flipped to avoid condensation on the agar. To store the plates, parafilm was used to wrap around the edge between the plate and the cover.

### 2.13. Antibacterial Test

In this test, two environmental conditions were evaluated: dark and ultra-violet (UV) light. The measurements were conducted in triplicate, using the procedure as follows: A 2.0 cm^2^ film coupon was soaked in 8.0 mL of bacterial solution for 2 h in a petri dish. Under the dark condition, the petri dish was put in a small incubator, of which window was covered with aluminum foil. Under the UV-light condition, the petri dish was placed in a biosafety cabinet equipped with the UV setting at room temperature. After each treatment, 1.0 mL of the tested bacterial solution was taken out, and five 10-fold dilutions were carried out. 200 μL of the highest dilution was spread on an agar plate with a cell spreader. The agar plates were incubated at 37 °C for 24 h in the dark, where the colony-forming units (CFU) were counted afterward.

## 3. Results and Discussion

### 3.1. Preparation of Ag/TiO_2_/Cellulose Composite Films

**Figure 1:**
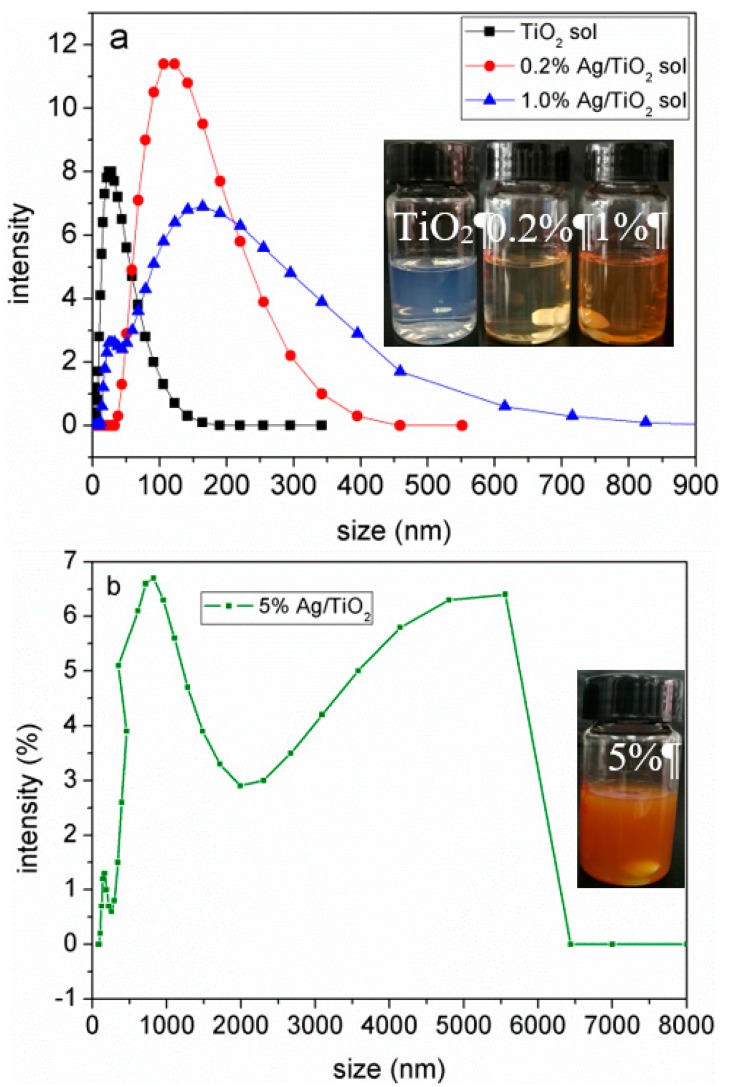
Dynamic light scattering (DLS) spectra of the TiO_2_ sol and TiO_2_/Ag sols with different Ag content.

A simple and green method based on colloid chemistry was demonstrated to prepare Ag/TiO_2_/cellulose composite films at room temperature. In this method, ascorbic acid (i.e., vitamin C) was chosen as the reducing agent due to its non-toxic and mild reducing ability (in contrast with the commonly used and more toxic NaBH_4_), which resulted in a homogeneous dispersion of Ag NPs among the continuous TiO_2_ thin film deposited on the surface of cellulose paper. With this method, the zeta potential of the TiO_2_ colloidal suspension was found to be +36.9 mV. Such a large charge value minimized the tendency of particle agglomeration due to electrostatic repulsion, thus promoting the stability of the TiO_2_ sol. The size distribution of the TiO_2_ hydrosol was determined by dynamic light scattering (DLS, Malvern Panalytical Ltd, Malvern, UK), where the average size was about 21 nm. As for the Ag/TiO_2_ sol with different Ag added, the zeta potentials were found to be +29.8, +28.6, and +26.4 mV, respectively. These results indicate that the addition of Ag reduced the stability of the TiO_2_ sol; however, the mixture was still relatively stable at least for 5 h at room temperature. It was seen that DLS of the Ag/TiO_2_ sol exhibited a broader distribution with the increase of Ag concentration (Figure 1a). With the highest Ag concentration (5%), the Ag/TiO_2_ sol showed a bimodal distribution due to the large Ag NPs aggregation (Figure 1b).

### 3.2. Structure and Morphology Characterization of Ag/TiO_2_/cellulose Composite Films

**Figure 2:**
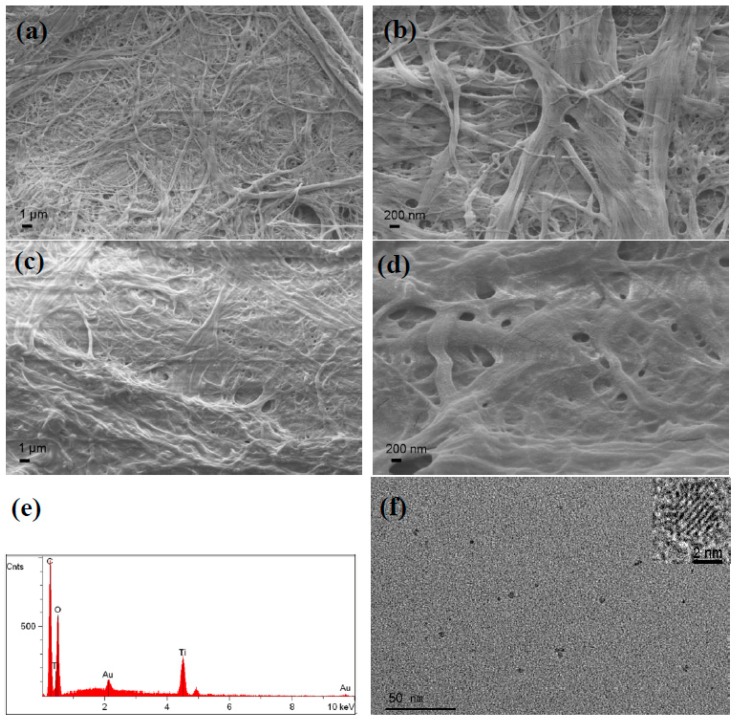
Scanning electron microscope (SEM) images of cellulose paper (substrate) at lower magnification (**a**) and higher magnification (**b**); the TiO_2_/cellulose composite film at lower magnification (**c**) and higher magnification (**d**); energy-dispersive spectroscopy (EDS) elemental analysis of TiO_2_/cellulose composite film (**e**); transmission electron microscope (TEM) of TiO_2_ particles peeled off from TiO_2_/cellulose film by vigorous stirring, followed by sonication (**f**).

Figure 2 shows the SEM images of cellulose paper (substrate), TiO_2_/cellulose composite film (without Ag), and EDS elemental analysis of the composite film, respectively. Compared to the base cellulose paper (Figure 2a,b), the TiO_2_/cellulose composite shows an evenly distributed and continuous TiO_2_ thin film layer (Figure 2c,d), while preserving the original microfiber structures of the filter paper. The higher magnification images show that most of the hierarchical fibers on the surface of the cellulose paper are uniformly coated with the TiO_2_ layer (Figure 2d). The EDS analysis (Figure 2e) confirmed the presence of the Ti element, with major peaks occurring at 4.51, 4.93, and 0.45 eV, indicating the formation of TiO_2_. Due to the formation of a continuous, dense film layer, it was difficult to observe individual TiO_2_ nanoparticles. To reveal the structure of TiO_2_ NPs, a piece of TiO_2_/cellulose film was vigorously stirred in water to peel off the TiO_2_ layer, which was subsequently sonicated. The TEM image of the resulting TiO_2_ sample is shown in Figure 2f, which indicated that the size of the TiO_2_ NPs was in the range of 3–5 nm. High-resolution TEM imaging of this sample was also carried out, where the lattice space of 0.35 nm indicated that the TiO_2_ NPs had the anatase phase (the inset of Figure 2f).

**Figure 3:**
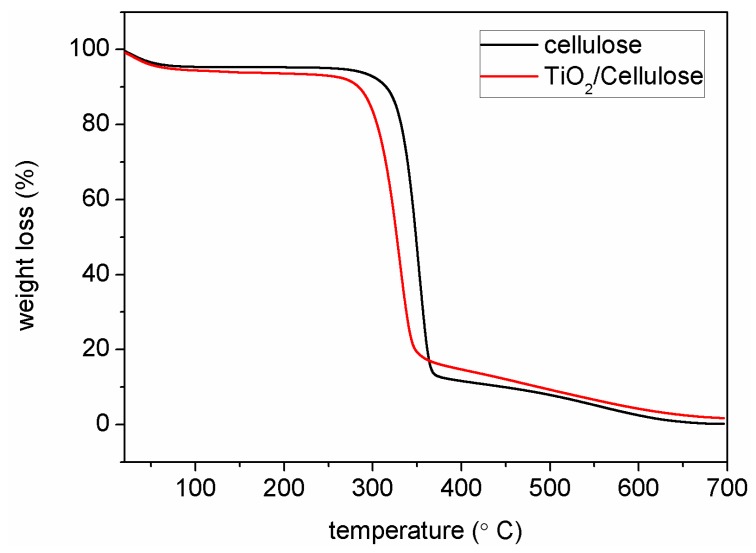
Thermal gravimetric analysis (TGA) of cellulose paper and TiO_2_/cellulose composite film.

The TGA analysis was conducted to determine the TiO_2_ content in the TiO_2_/cellulose composite film. The results are illustrated in Figure 3. It was found that the TiO_2_/cellulose film underwent three weight-loss stages. The first stage appeared below 100 °C, which could be attributed to the loss of physically adsorbed water. The second stage appeared in the range of 250–350 °C, which could be attributed to the carbonization process by dehydration, depolymerization, and decomposition of the cellulose substrate. The third stage appeared between 350 and 700 °C, which could be attributed to the complete decomposition of the cellulose substrate, where the residue was the inorganic component of TiO_2_. Based on this technique, the content of TiO_2_ determined by TGA was found to be 1.74 wt % (Figure 3).

For the Ag/TiO_2_/cellulose composite films, the Ag content could not be determined from the TGA technique because both Ag and TiO_2_ would remain after cellulose decomposition. To overcome this problem, the Ag^+^ concentration was determined by the inductively coupled plasma-optical emission spectrometry (ICP-OES, Thermo Icap 6300, Thermo Scientific, Waltham, MA, USA) method through acid digestion of the Ag/TiO_2_/cellulose composite films. As a result, the Ag weight percentage in the Ag/TiO_2_/cellulose films were calculated to be 0.003, 0.009, and 0.030 wt %, corresponding to the theoretical addition of 0.2%, 1.0%, and 5% molar ratio of Ag to TiO_2_.

**Figure 4:**
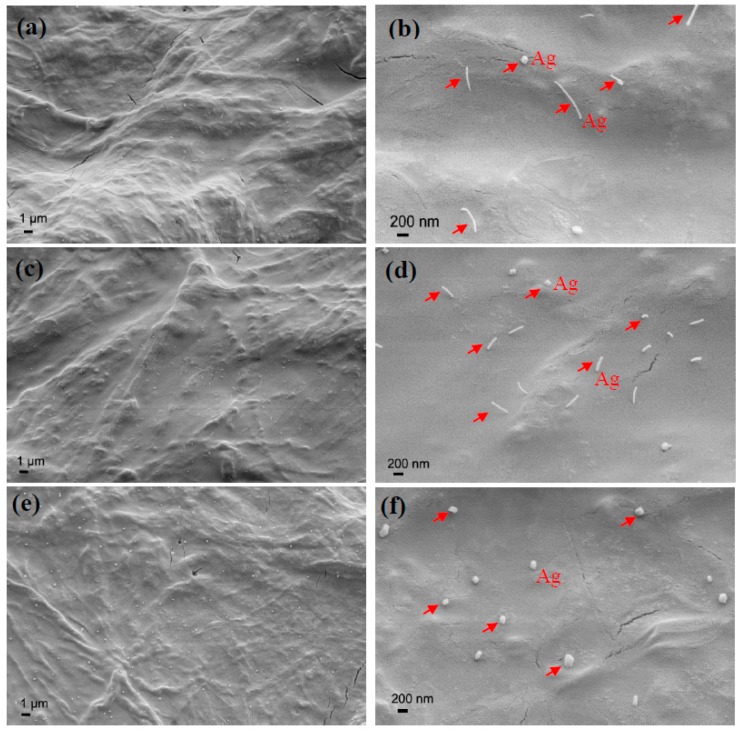
SEM images of Ag/TiO_2_/cellulose composite films with different loadings of Ag at lower and higher magnification: (**a**,**b**) 0.2 mol % ratio of Ag to TiO_2_; (**c**,**d**) 1.0 mol % ratio of Ag to TiO_2_; and (**e**,**f**) 5.0 mol % ratio of Ag to TiO_2_.

Figure 4 shows the surface morphology of the varying Ag/TiO_2_/cellulose composite films. It was found that Ag NPs were dispersed uniformly in the TiO_2_ layer without aggregation. Interestingly, Ag NPs exhibited the form of nanowire (the diameter was between 30–50 nm), which were clearly seen in Figure 4b,d at the lower ratios of Ag (i.e., 0.2 and 1.0 mol %). At the higher ratio of Ag (5.0 mol %), the diameter of Ag NPs was found to increase quite substantially, and were in the range of 100–200 nm. Perhaps this indicates that at low Ag concentration, the NP was dominated by the 1D crystal growth, leading to a nanowire morphology; where at higher Ag concentration (e.g., 5 mol %), the NP possessed the 3D crystal growth, leading to the greatly increased diameter. It has been noted that the ascorbic acid could act not only as a reducing agent, but also as a stabilizing agent in this sol–gel process [42].

**Figure 5:**
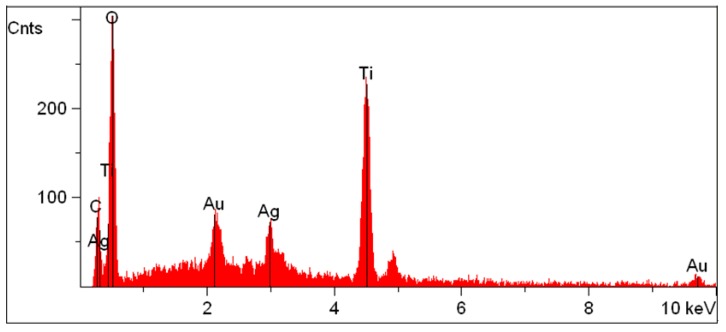
EDS analysis of Ag/TiO_2_/cellulose composite film doped with 5 mol % of Ag.

The EDS spectra did not reveal any Ag signals in the Ag/TiO_2_/cellulose composite films at the lower molar ratios of Ag (0.2–1.0 mol %) because the Ag content was very low. However, for the sample with 5 mol % of Ag/TiO_2_, the Ag signal occurring around 3.0 keV was clearly observed, as shown in Figure 5.

**Figure 6:**
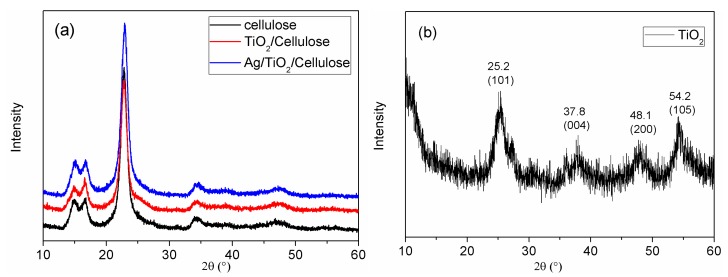
X-ray diffraction (XRD) pattern for (**a**) cellulose paper, TiO_2_/cellulose, and Ag/TiO_2_/cellulose composite film; (**b**) the Ag/TiO_2_ powder peeled off the composite showing the distinct crystalline phase of TiO_2_.

The XRD patterns of cellulose, TiO_2_/cellulose, and Ag/TiO_2_/cellulose composite films and TiO_2_ powder are shown in Figure 6. In Figure 6a, it can be seen that all three diffraction profiles are similar, dominated by the cellulose diffraction peaks. This is reasonable because of the low content of TiO_2_ and Ag in the composite films. To determine the crystal structure of TiO_2_ NPs, Ag/TiO_2_ powder was peeled off from the cellulose substrate by vigorously stirring the Ag/TiO_2_/cellulose film in water. In Figure 6b, it can be seen that the peeled Ag/TiO_2_ powder shows a distinct crystalline phase of TiO_2_ with no signs of Ag crystals due to its very small doping amount. The diffraction peaks for the TiO_2_ powder located at 2θ = 25.2°, 37.8°, 48.1°, and 54.2° could be indexed by the (101), (004), (200), and (105) diffraction peaks of the TiO_2_ anatase phase, respectively, which are consistent with the TEM results. We note that anatase is generally recognized to be the most active among the common crystal phases of TiO_2_ [43,44,45].

### 3.3 Antibacterial Activities

**Figure 7:**
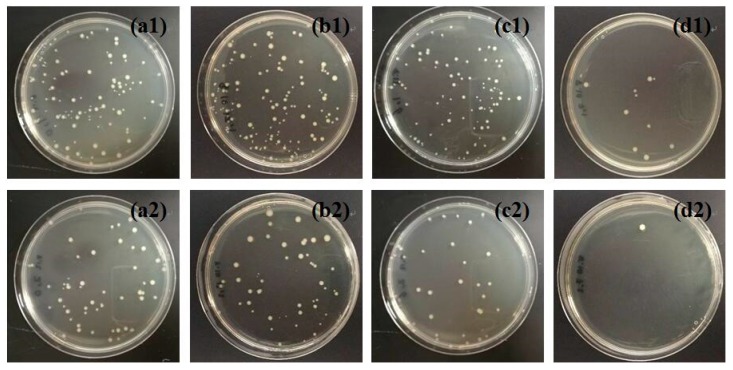
Dark-condition antibacterial results for (**a1**) control; (**b1**) cellulose; (**c1**) TiO_2_/cellulose composite film; and (**d1**) Ag/TiO_2_/cellulose composite film (Ag/TiO_2_ molar ratios of 5.0%). Ultra-violet (UV)-condition antibacterial results for (**a2**) control; (**b2**) cellulose; (**c2**) TiO_2_/cellulose composite film; and (**d2**) Ag/TiO_2_/cellulose composite film (Ag/TiO_2_ molar ratios of 5.0%).

**Figure 8:**
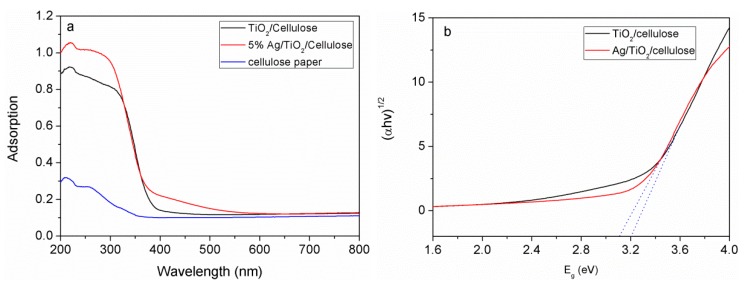
(**a**) UV-vis diffuse reflectance spectra (DRS) of cellulose filter paper; TiO_2_/cellulose; and Ag/TiO_2_/cellulose film. (**b**) The band gap of TiO_2_/cellulose and Ag/TiO_2_/cellulose film.

The antibacterial activities of TiO_2_/cellulose and Ag/TiO_2_/cellulose composite films were evaluated against *E. coli* under dark and UV conditions. For comparison, pristine cellulose filter paper was also tested under the same conditions, and the results are shown in Figure 7. It can be seen from Figure 7b1,b2 that the number of CFU increased by approximately 20% in the dark condition, when compared to the control group without the addition of cellulose. This indicates that the cellulose substrate is prone to bacteria growth. TiO_2_/cellulose composite films were found to have little antibacterial effects under either of the dark or UV conditions (Figure 7c1,c2). It was seen that Ag/TiO_2_/cellulose composites containing 5 mol % Ag/TiO_2_ displayed significant antibacterial activity against *E. coli*, where almost all *E. coli* were inhibited under the UV condition (Figure 7d1,d2). Compared to the TiO_2_/cellulose composite film, Ag/TiO_2_/cellulose composite film exhibited superior antibacterial performance against *E. coli* due to the synergetic effect of silver and anatase TiO_2_, which can be explained as follows. Under UV irradiation, TiO_2_ nanocrystals can effectively generate ROS, such as hydroxyl radicals (OH) and other reactive oxygen species, including superoxide anion (O_2_^−^) and hydrogen peroxide (H_2_O_2_). The ROS can interact with the cell wall through chemical binding, thus inactivating the phosphorus species and eventually causing bacterial death [46]. With the additional doping of Ag NPs, Ag NPs act as electron traps, and the electron transferring from TiO_2_ to Ag can further inhibit the recombination of photon-generated electron/hole pairs, as confirmed by the red shift of light adsorption in UV-vis diffuse reflectance spectra (DRS) and its estimated decreased band gap (as shown in Figure 8), which promoted the formation of more ROS. As a result, the antibacterial activity of Ag/TiO_2_/cellulose was significantly improved. In addition, the good dispersion of Ag NPs could enhance the surface area to the mass ratio that might favor the direct transfer from the chemisorbed silver ions in the Ag/TiO_2_/cellulose to the bacteria upon contact, thus further enhancing the biocidal effect [19].

**Figure 9:**
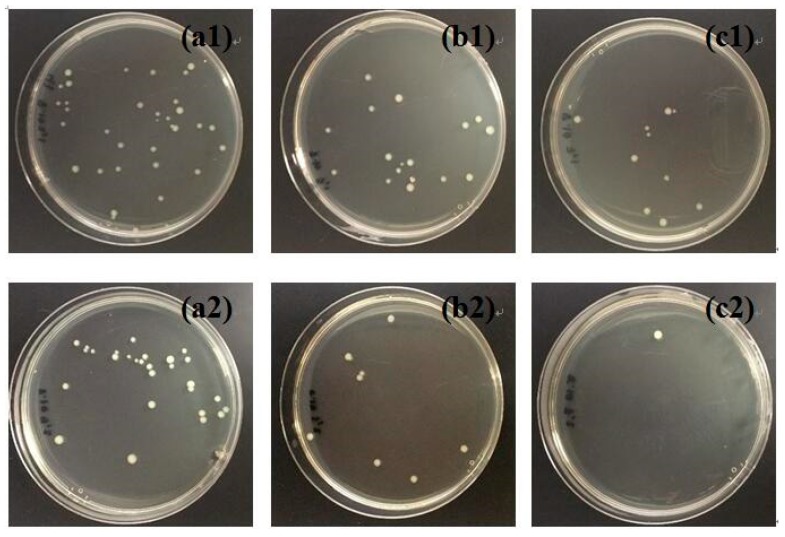
Antibacterial results under the dark condition for Ag/TiO_2_/cellulose composite films with Ag/TiO_2_ molar ratios of (**a1**) 0.2%; (**b1**) 1.0%; and (**c1**) 5.0%. Antibacterial results under the UV condition for Ag/TiO_2_/cellulose composite films with Ag/TiO_2_ molar ratios of (**a2**) 0.2%; (**b2**) 1.0%; and (**c2**) 5.0%.

**Figure 10:**
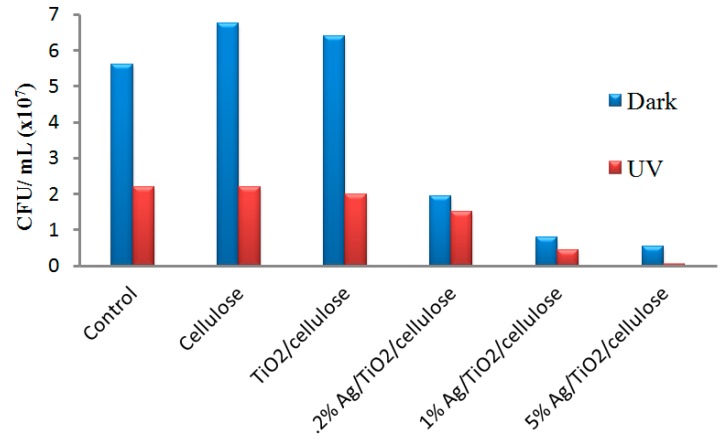
Bar chart of the antibacterial effect of cellulose; TiO_2_/cellulose; Ag/TiO_2_/cellulose composite films with Ag/TiO_2_ molar ratios of 0.2%; 1%; and 5% under dark and UV light.

Figure 9 illustrates the antibacterial results under dark and UV conditions for Ag/TiO_2_/cellulose composite films with Ag/TiO_2_ molar ratios of (Figure 9a1) 0.2%, (Figure 9b1) 1.0%, and (Figure 9c1) 5.0%, respectively. It was found that the antibacterial activity against *E. coli* was greatly enhanced with an increase in the Ag doping content. The incorporation of 5 mol % Ag/TiO_2_ nanocomposites onto cellulose filter paper appeared to inhibit almost all bacteria colonies under the UV condition. The CFU were counted from both Figure 7 and Figure 9, where the results, in the form of a bar chart, are illustrated in Figure 10. It was apparent that the antibacterial activity of the Ag/TiO_2_/cellulose composite film outperformed all other samples, where 5 mol % Ag/TiO_2_ was able to inhibit more than 99% of *E. coli* under the UV condition.

## 4. Conclusions

Ag/TiO_2_/cellulose nanocomposite films were fabricated by a sol–gel method at room temperature. In this method, AgNO_3_ was first added into a TiO_2_ sol, and Ag nanocrystals were generated in situ by ascorbic acid (i.e., vitamin C). The method is green, simple, and easy to scale up. The synergistic effects of the uniform coating of anatase TiO_2_ nanocrystals (in the form of granules with a diameter ranging from 3–5 nm) and the incorporated, well-dispersed Ag nanocrystals enhanced the antibacterial activity of the resulting Ag/TiO_2_/cellulose nanocomposite films. The inclusion of 5% molar ratios of Ag/TiO_2_ in these composite films exhibited the best antibacterial performance against *E. coli,* where more than 99% of *E. coli* were inhibited under the UV condition. The demonstrated Ag/TiO_2_/cellulose composite system has great potential for practical antibacterial applications in both healthcare and water purification industries.

## References

[1] Klemm D., Heublein B., Fink H.P., Bohn A. (2005). Cellulose: Fascinating biopolymer and sustainable raw material. Angew. Chem. Int. Ed..

[2] Moon R.J., Martini A., Nairn J., Simonsen J., Youngblood J. (2011). Cellulose nanomaterials review: Structure, properties and nanocomposites. Chem. Soc. Rev..

[3] Ma H.Y., Burger C., Hsiao B.S., Chu B. (2011). Ultrafine Polysaccharide Nanofibrous Membranes for Water Purification. Biomacromolecules.

[4] Mohammed N., Grishkewich N., Tam K.C. (2018). Cellulose nanomaterials: Promising sustainable nanomaterials for application in water/wastewater treatment processes. Environ. Sci. Nano.

[5] Carpenter A.W., de Lannoy C.F., Wiesner M.R. (2015). Cellulose Nanomaterials in Water Treatment Technologies. Environ. Sci. Technol..

[6] Cheng Q.Y., Ye D.D., Yang W.T., Zhang S.H., Chen H.Z., Chang C.Y., Zhang L.N. (2018). Construction of Transparent Cellulose-Based Nanocomposite Papers and Potential Application in Flexible Solar Cells. ACS Sustain. Chem. Eng..

[7] Shchipunov Y., Postnova I. (2018). Cellulose Mineralization as a Route for Novel Functional Materials. Adv. Funct. Mater..

[8] Jia C., Li T., Chen C.J., Dai J.Q., Kierzewski I.M., Song J.W., Li Y.J., Yang C.P., Wang C.W., Hu L.B. (2017). Scalable, anisotropic transparent paper directly from wood for light management in solar cells. Nano Energy.

[9] Wang X.D., Yao C.H., Wang F., Li Z.D. (2017). Cellulose-Based Nanomaterials for Energy Applications. Small.

[10] Peng Z.Y., Zou Y.B., Xu S.Q., Zhong W.B., Yang W.T. (2018). High-Performance Biomass-Based Flexible Solid-State Supercapacitor Constructed of Pressure-Sensitive Lignin-Based and Cellulose Hydrogels. ACS Appl. Mater. Interf..

[11] Bu Y., Cao M.L., Jiang Y.Y., Gao L., Shi Z.J., Xiao X., Wang M.K., Yang G., Zhou Y.H., Shen Y. (2018). Ultra-thin bacterial cellulose/poly(ethylenedioxythiophene) nanofibers paper electrodes for all-solid-state flexible supercapacitors. Electrochim. Acta.

[12] Neely A.N., Maley M.P. (2000). Survival of enterococci and staphylococci on hospital fabrics and plastic. J. Clin. Microbiol..

[13] Iyigundogdu Z.U., Demir O., Asutay A.B., Sahin F. (2017). Developing Novel Antimicrobial and Antiviral Textile Products. Appl. Biochem. Biotechnol..

[14] Rai M., Yadav A., Gade A. (2009). Silver nanoparticles as a new generation of antimicrobials. Biotechnol. Adv..

[15] Morones J.R., Elechiguerra J.L., Camacho A., Holt K., Kouri J.B., Ramirez J.T., Yacaman M.J. (2005). The bactericidal effect of silver nanoparticles. Nanotechnology.

[16] Richard L.D., Samuel F.E. (1997). The development and functions of silver in water purification and disease control. Catal. Today.

[17] Dankovich T.A., Gray D.G. (2011). Bactericidal paper impregnated with silver nanoparticles for point-of-use water treatment. Environ. Sci. Technol..

[18] Park S.-H., Ko Y.-S., Park S.-J., Lee J.S., Cho J., Baek K.-Y., Kim I.T., Woo K., Lee J.-H. (2016). Immobilization of silver nanoparticle-decorated silica particles on polyamide thin film composite membranes for antibacterial properties. J. Membr. Sci..

[19] Regiel A., Irusta S., Kyziol A., Arruebo M., Santamaris J. (2013). Preparation and characterization of chitosan-silver nanocomposite films and their antibacterial activity against staphylococcus aureus. Nanotechnology.

[20] Daoud W.A., Xin J.H., Zhang Y.-H. (2005). Surface functionalization of cellulose fibers with titanium dioxide nanoparticles and their combined bactericidal activities. Surf. Sci..

[21] Chauhan I., Mohanty P. (2014). In situ decoration of TiO_2_ nanoparticles on the surface of cellulose fibers and study of their photocatalytic and antibacterial activities. Cellulose.

[22] Abdel Rehim M.H., El-Samahy M.A., Badawy A.A., Mohram M.E. (2016). Photocatalytic activity and antimicrobial properties of paper sheets modified with TiO_2_/Sodium alginate nanocomposites. Carbohydr. Polym..

[23] Luo Y., Huang J.G. (2015). Hierarchical-Structured Anatase-Titania/Cellulose Composite Sheet with High Photocatalytic Performance and Antibacterial Activity. Chemistry.

[24] Perkas N., Lipovsky A., Amirian G., Nitzan Y., Gedanken A. (2013). Biocidal properties of TiO_2_ powder modified with Ag nanoparticles. J. Mater. Chem. B.

[25] Pan X., Medina-Ramirez I., Mernaugh R., Liu J. (2010). Nanocharacterization and bactericidal performance of silver modified titania photocatalyst. Colloid Surf. B.

[26] Abdel-Fatah W.I., Gobara M.M., Mustafa S.F.M., Ali G.W., Guirguis O.W. (2016). Role of silver nanoparticles in imparting antimicrobial activity of titanium dioxide. Mater. Lett..

[27] Thiel J., Pakstis L., Buzby S., Raffi M., Ni C., Pochan D.J., Shah S.I. (2007). Antibacterial properties of silver-doped titania. Small.

[28] Cacciato G., Bayle M., Pugliara A., Bonafos C., Zimbone M., Privitera V., Grimaldi M.G., Carles R. (2015). Enhancing carrier generation in TiO_2_ by a synergistic effect between plasmon resonance in Ag nanoparticles and optical interference. Nanoscale.

[29] Ali T., Ahmed A., Alam U., Uddin I., Tripathi P., Muneer M. (2018). Enhanced photocatalytic and antibacterial activities of Ag-doped TiO_2_ nanoparticles under visible light. Mater. Chem. Phys..

[30] Hussain M., Tariq S., Ahmad M., Sun H.Y., Maaz K., Ali G., Hussain S.Z., Iqbal M., Karim S., Nisar A. (2016). Ag-TiO_2_ nanocomposite for environmental and sensing applications. Mater. Chem. Phys..

[31] Liu H., Dong X.N., Nan L., Ma H.X., Chen X.J., Zhu Z.F. (2015). A novel fabrication of silver-modified TiO_2_ colloidal-assembled microstructures and enhanced visible photocatalytic activities. Mater. Lett..

[32] Xu H.F., Li G., Liu N., Zhu K.R., Zhu G., Jin S.W. (2015). Ag @ hierarchical TiO_2_ core-shell nanostructures for enhanced photocatalysis. Mater. Lett..

[33] Zhang F.L., Cheng Z.Q., Cui L.Y., Duan T.T., Anan A., Zhang C.F., Kang L.J. (2016). Controllable synthesis of Ag@TiO_2_ heterostructures with enhanced photocatalytic activities under UV and visible excitation. RSC Adv..

[34] Wang D., Zhou Z.-H., Yang H., Shen K.-B., Huang Y., Shen S. (2012). Preparation of TiO_2_ loaded with crystalline nano Ag by a one-step low-temperature hydrothermal method. J. Mater. Chem..

[35] Yu D.H., Yu X., Wang C., Liu X.C., Xing Y. (2012). Synthesis of natural cellulose-templated TiO_2_/Ag nanosponge composites and photocatalytic properties. ACS Appl. Mater. Interfaces.

[36] Ginter J., Kisielewska A., Spilarewicz-Stanek K., Cichomski M., Batory D., Piwonski I. (2016). Tuning of the photocatalytic activity of thin titanium dioxide coatings by highly ordered structure and silver nanoparticles. Microporous Mesoporous Mater..

[37] Mahy J.G., Lambert S.D., Leonard G.L.M., Zubiaur A., Olu P.Y., Mahmoud A., Boschini F., Heinrichs B. (2016). Towards a large scale aqueous sol–gel synthesis of doped TiO_2_: Study of various metallic dopings for the photocatalytic degradation of p-nitrophenol. J. Photochem. Photobiol. A.

[38] Li S., Huang J.G. (2016). Cellulose-Rich Nanofiber-Based Functional Nanoarchitectures. Adv. Mater..

[39] Cai H., Mu W., Liu W., Zhang X., Deng Y. (2015). Sol–gel synthesis highly porous titanium dioxide microspheres with cellulose nanofibrils-based aerogel templates. Inorg. Chem. Commun..

[40] Galkina O.L., Sycheva A., Blagodatskiy A., Kaptay G., Katanaev V.L., Seisenbaeva G.A., Kessler V.G., Agafonov A.V. (2014). The sol–gel synthesis of cotton/TiO_2_ composites and their antibacterial properties. Surf. Coat. Technol..

[41] Daoud W.A., Xin J.H. (2004). Nucleation and Growth of Anatase Crystallites on Cotton Fabrics at low temperature. J. Am. Ceram. Soc..

[42] Ludivine M., Rémi D., Ryan J.M., Lawrence A.H., Bertrand D., Christopher B.M. (2016). One-step green synthesis of gold and silver nanoparticles with ascorbic acid and their versatile surface post-functionalization. RSC Adv..

[43] Pantaroto H.N., Ricomini A.P., Bertolini M.M., da Silva J.H.D., Neto N.F.A., Sukotjo C., Rangel E.C., Barao V.A.R. (2018). Antibacterial photocatalytic activity of different crystalline TiO_2_ phases in oral multispecies biofilm. Dent. Mater..

[44] Li W., Bai Y., Liu C., Yang Z., Feng X., Lu X., van der Laak N.K., Chan K.-Y. (2009). Highly Thermal Stable and Highly Crystalline Anatase TiO_2_ for Photocatalysis. Environ. Sci. Technol..

[45] Tanaka K., Capule M.F.V., Hisanaga T. (1991). Effect of crystallinity of TiO_2_ on its photocatalytic action. Chem. Phys. Lett..

[46] Shuai C., Shuai C., Feng P., Gao C., Peng S., Yang Y. (2018). Antibacterial Capability, Physicochemical Properties, and Biocompatibility of nTiO_2_ Incorporated Polymeric Scaffolds. Polymers.

